# The Impact of COVID-19 Management Policies Tailored to Airborne SARS-CoV-2 Transmission: Policy Analysis

**DOI:** 10.2196/20699

**Published:** 2021-04-21

**Authors:** Charles Roberto Telles, Archisman Roy, Mohammad Rehan Ajmal, Syed Khalid Mustafa, Mohammad Ayaz Ahmad, Juan Moises de la Serna, Elisandro Pires Frigo, Manuel Hernández Rosales

**Affiliations:** 1 Internal Control Center Secretary of State for Education and Sport of Paraná Curitiba Brazil; 2 Mathematics Group Department of Physics, Faculty of Science Institute of Science, Banaras Hindu University Varanasi India; 3 Department of Biochemistry Faculty of Science University of Tabuk Tabuk Saudi Arabia; 4 Department of Chemistry Faculty of Science University of Tabuk Tabuk Saudi Arabia; 5 Department of Physics Faculty of Science University of Tabuk Tabuk Saudi Arabia; 6 Universidad Internacional de la Rioja Madrid Spain; 7 Federal University of Paraná Curitiba Brazil; 8 Programa Universitario de Estudios Sobre la Ciudad National Autonomous University of Mexico Mexico City Mexico

**Keywords:** social distancing policies, COVID-19, airborne transmission, convergence and stability properties

## Abstract

**Background:**

Daily new COVID-19 cases from January to April 2020 demonstrate varying patterns of SARS-CoV-2 transmission across different geographical regions. Constant infection rates were observed in some countries, whereas China and South Korea had a very low number of daily new cases. In fact, China and South Korea successfully and quickly flattened their COVID-19 curve. To understand why this was the case, this paper investigated possible aerosol-forming patterns in the atmosphere and their relationship to the policy measures adopted by select countries.

**Objective:**

The main research objective was to compare the outcomes of policies adopted by countries between January and April 2020. Policies included physical distancing measures that in some cases were associated with mask use and city disinfection. We investigated whether the type of social distancing framework adopted by some countries (ie, without mask use and city disinfection) led to the continual dissemination of SARS-CoV-2 (daily new cases) in the community during the study period.

**Methods:**

We examined the policies used as a preventive framework for virus community transmission in some countries and compared them to the policies adopted by China and South Korea. Countries that used a policy of social distancing by 1-2 m were divided into two groups. The first group consisted of countries that implemented social distancing (1-2 m) only, and the second comprised China and South Korea, which implemented distancing with additional transmission/isolation measures using masks and city disinfection. Global daily case maps from Johns Hopkins University were used to provide time-series data for the analysis.

**Results:**

The results showed that virus transmission was reduced due to policies affecting SARS-CoV-2 propagation over time. Remarkably, China and South Korea obtained substantially better results than other countries at the beginning of the epidemic due to their adoption of social distancing (1-2 m) with the additional use of masks and sanitization (city disinfection). These measures proved to be effective due to the atmosphere carrier potential of SARS-CoV-2 transmission.

**Conclusions:**

Our findings confirm that social distancing by 1-2 m with mask use and city disinfection yields positive outcomes. These strategies should be incorporated into prevention and control policies and be adopted both globally and by individuals as a method to fight the COVID-19 pandemic.

## Introduction

### Unexpected Forms of Transmission and the Role of Policy

The COVID-19 pandemic consistently demonstrated a pattern of growing community transmission worldwide, even with the adoption of social distancing measures (lockdown or voluntarily shelter in place) in January and early May 2020. The continuing transmission of the virus despite the policy measures adopted in some countries was an important point of debate and investigation in the scientific community and among authorities. Unexpected forms of transmission (atmospheric [1-3]) associated with the social distancing policy became the central question for the infectious transmission modeling of SARS-CoV-2 and predictive methods.

This research considers the advanced phases of community transmission observed in some countries [4] in a select period. Due to the increasing numbers of new infections and deaths, monitored by the World Health Organization [4] and Johns Hopkins University, this research is mainly focused on the nonlinear epidemic properties of SARS-CoV-2 transmission. These nonlinear epidemic properties of transmission can be understood through the highly random forms of virus transmission associated with human social behavior and with environmental conditions (physical or aerosol long-range transmission, airborne transmission). In this research, nonlinearity refers mainly to the unpredictability of the epidemiologic framework of the SIR (susceptible, infected, removed) stochastic models used to track the possible rate of infection in the population, even with some policy measures implemented by countries [5-8]. This limited ability to predict future rates of contagion was noted during the spread of the pandemic. It was suggested that the qualitative theory of differential equations may be appropriate for identifying the variables, policies, or environmental conditions that influence the constant propagation of the virus. The random patterns of virus reproduction suggest that transmission happens through the air. Other dimensions of research must be considered—the social behavior of individuals and the aerosol fluid dynamic behavior. This direction of research has yielded unresolved mathematical equations that simulate the daily growth of new cases. This study defined the aerosol, or biosol, or ground form of transmission as spreading patterns of infection. The policy measure adopted by a country may or may not address these spreading patterns adequately, which then may sustain (or not) dissemination patterns of the virus worldwide. In this way, the spreading pattern is related to the forms of virus transmission. At the same time, the dissemination of the virus, regardless of how it can be transmitted, depends on the cultural, personal, and policy aspects of managing societal and individual behaviors.

In this study, geographical regions in Asia, South America, North America, the Middle East, Africa, and Europe were analyzed to confirm whether the policies adopted by China and South Korea during the outbreak were the most effective ones in the period of January to April 2020. During this period, only these two countries had adopted specific policy measures addressing the airborne framework of SARS-CoV-2 transmission beyond social distancing (mask wearing and city disinfection). These countries also had the lowest daily new case counts of COVID-19. The relationship between mask wearing, city disinfection, and the airborne form of transmission during the period of interest will be used to test the hypothesis that the virus can be transmitted through the air.

### Theoretical Analysis of the Nonlinear Properties of SARS-CoV-2 Dissemination Patterns

SARS-CoV-2 follows different patterns of transmission among humans [5-7]. These patterns are being investigated not only using clinical trials, statistical tools [5-11], and medical interviews with patients [9,10], but also from a mathematical point of view, using SIR compartmental models with a high degree of uncertainty. Concerning mathematical predictions of SARS-CoV-2 reproductive patterns within a complex network of human behavior [5], the maximum possible rate of infection with the virus in daily human life [5-8,12,13] consists of a community dissemination pattern with an increasing margin of statistically unpredictable outcomes. The models were still being developed due to predictive failures. One specific unpredictable pattern [14] of the virus spread and dissemination from January to April 2020 is visible in the numbers of new infections over time in countries where the input and output (which is the number of people who could be infected from an initial number, resulting in maximum and minimum margins of dissemination of the virus fluctuation) expressed unpredictability. This observation was initially and briefly modeled by Koerth et al [15].

Regarding these nonlinear aspects of infection within countries, this study points out that there is evidence for long-range airborne transmission [16-18] of SARS-CoV-2. The evidence consists of the type of policies adopted in China and South Korea from January to April 2020, where a significant reduction in infection cases occurred, with distinct patterns found in other countries during all epidemic contagion phases. China and South Korea instituted social or physical distancing measures along with additional methods, such as mask use and city disinfection. It was one of the main causes of the nontrivial frequency of daily new COVID-19 case distribution during the early stage of the pandemic, up to late April and early May. Physical distancing with an air preventive framework was revealed to be an urgent need for any country at that time, and, along with social distancing and testing policies, is now one of the main preventive methods used.

Recent studies reported that the transmission of SARS-CoV-2 occurs due to proximity to other humans and to social interactions within a set of empirical variables, including the most basic forms of human behavior, such as coughing, sneezing, handshakes, sharing clothes, sharing cups, general touching, and general object-sharing behaviors [19,20]. This set of variables influences transmission, together with the environmental factors associated with the virus’s possible transmission on the ground (surfaces) and in the air (not only aerosols in medical facilities but aerosol and biosol formed under atmospheric conditions outdoors). This leads to new patterns for course epidemiology [12]. Between January and April 2020, the World Health Organization confirmed aerosol transmission only at medical facilities [21], not in outdoor urban spaces. However, van Doremalen et al [22] stated early on that human upper and lower respiratory tracts cause the nearby atmosphere to become infected, propagating the virus through the air. They measured this effect for about 3 hours during an experiment and observed low infection reduction over time, with infectious titer changing from 10^3.5^ to 10^2.7^ TCID_50_ (50% tissue culture infective dose) for SARS-CoV-2 [22]. An alternative scientific hypothesis and further probabilistic and statistical frameworks were needed to establish new policies and guide individual preventive actions. Although a scientific breakthrough occurred early in the pandemic, no policy measure was announced as definitive, and each country was searching for preventive methods independently. This is why it is worthwhile to compare how some countries reduced SARS-CoV-2 transmission with specific social distancing measures.

The analysis of the nonlinear properties of the mathematical models and nonpharmaceutical interventions for the COVID-19 epidemiological framework is important not only for medical facilities but also for public policies and health care infrastructure. It can help to estimate the disease patterns of community transmission in a pandemic scenario that affect the economy and threaten people’s health and survival. This research is also relevant due to the large active workforce trying to maintain essential services and sectors necessary for survival, such as electrical, water, garbage disposal, energy, food supply/production, commerce, and industry.

### COVID-19 Transmission Instability

Policy that consists of physical distance between individuals may fail because the virus may continue to be transmitted in other unexpected ways. This instability becomes visible when countries that adopt this policy still fail to contain virus spread due to asymptotic instability between the virus’s potential to infect individuals in spite of the policy measures and methodology. The unbalancing of this equation is found in a wide variety of probability distributions of daily new cases, with distinct patterns [6-9,12,13,15,19,23] observed in many countries [4]. This may be why new cases continued to occur between January and April 2020, even with preventive methods such as social distancing (lockdown or shelter in place) and COVID-19 testing.

Causes beyond the traditional transmission analysis [5-9,13,24-26] need to be considered to explain the continued growth of new cases. Other factors for transmission and modeling patterns should be considered and constructed [12,13,15,27-30] using mathematical counterproof predictions for countries that had already adopted social distancing and had COVID-19 testing available but adopted social physical distancing measures with distinct parameters such as using or not using masks and city disinfection.

### Statistical Uncertainty and COVID-19 Prevention

Many variables affect virus transmission rates, such as the type of health policies adopted by each country, public health infrastructure, population genetics, human variance in biological resistance, local epidemic outbreaks, globalization aspects, COVID-19 testing availability, virus mutation, and citizens’ adherence to social physical distancing. The influence of these factors is visible on the Our World in Data webpage [31]. These confounding outcomes in each country make it difficult to determine why some countries still have an active virus infection and what would be the best fixed-point orientation (policy measure) to reduce virus transmission rates. However, worldwide statistical data can provide a relevant confidence interval analysis if different countries’ policies are compared. This would reveal the best approach for reducing virus infections. At the moment, policy is the most effective way to reduce COVID-19 cases since no vaccine or drugs have been consistently effective for treating the disease or stopping virus propagation worldwide.

Research shows that individual behavior and social ties [32-34] are still key for controlling the community transmission of the virus through social distancing measures. These measures must consider the dynamics of groups/communities and the community infrastructure (households, buses, shopping malls, meetings, markets, daily activities, and human behavior). Note that the term “social distancing” is used here to describe the behavior of an uninfected individual outside medical facilities and refers only to the population separation patterns based on ground distances. The term “social physical distancing” refers to one of the measures included in the social distancing policies.

To explain why the virus continues to be transmitted when social physical distancing is practiced, it is important to consider that social contact might still occur as a human physical connection during environmental socialization; that is, physical ground and atmospheric contact may occur. The policy requires individuals to stay 1 or 2 m apart, assuming that this is enough to prevent virus transmission, and has the same effect as sheltering in place (mandatory or not). However, with this measure, there are still many opportunities for social contact within a physical dimension at the ground and atmospheric levels, both indoors or outdoors, as observed in many studies [20,35-39].

We need to theoretically and empirically analyzed two parameters, social distancing policy and social transmission isolation, because environmental transmission may play a role in recurrent community transmission of SARS-CoV-2. The epidemiological methods of prediction and control (which are needed to estimate the supply of financial, economic, and public health resources for the predicted number of infected people) lose their effectiveness due to certain aspects of social transmission isolation and SARS-CoV-2’s airborne virulence potential [20,35-39]. This new approach diverges from older approaches, such as the one demonstrated by Hellewell et al [40], since social distancing and social transmission isolation parameters are different stages under atmospheric conditions, which require further empirical investigation.

Many recent viral infectious diseases (severe acute respiratory syndrome [SARS], Middle Eastern respiratory syndrome [MERS], H1N1) are transmitted similarly to SARS-CoV-2 [5], but they have different rates of exponential growth [41]. Therefore, it is important to consider not only the causes of transmission, such as the chemical and biological properties of transmission and the virus-human biological affinity but also the emergent virus and human social behavior in the context of the environment [35-40,42-47]. The nonlinear time series of worldwide policies may present a clue in the form of a high asymptotic stability (dissemination network) [37] about the type of preventive policy measures adopted by each country, as also observed previously by Riou and Althaus [48] with the *k* dispersion parameters and the superspreading prediction possibilities.

### Evidence for Airborne Transmission

The presence of these epidemiological factors (forms of transmission, biological-chemical affinities, and emergent social virus transmission behavior) associated with the preventive epidemic framework [49], implemented from January to April 2020, requires considering any given number of infected individuals as an ongoing pandemic threat, since uncertainty prevails. This led to the conclusion that there was no minimum range of infected individuals that would classify the local epidemic as under control. No policy adopted during the period of interest was more effective than those of China and South Korea. At that time, many authorities thought that the epidemic would have a natural upper limit and posterior descendant tail and would end naturally without any human intervention. However, it has not yet been scientifically proven that the pandemic can end naturally or become seasonal. Therefore, this theoretical observation should not have been used as a preventive measure at that time.

Concerning the evolution of the pandemic from January to April 2020, one important issue reported in the media is the difference between maintaining social physical distancing and full social isolation. Social physical distancing means maintaining physical distance in restaurants, parks, drugstore lines, household activities, neighborhoods (especially low-income neighborhoods), household tree proximity, markets, indoor and outdoor social events, windows and balconies, airplanes, ship balconies, hospital rooms, meetings, delivery or mail activities, prisons, residences, commercial establishments, and industrial facilities [50]. Full social transmission isolation, meanwhile, requires ground or atmospheric barriers. News and scientific reports [51,52] show that most of China and South Korea [51] had required residents to wear masks, and full disinfection had been implemented in crowded public spaces [15,53]. There had been some further concerns from public health professionals, as reported by Li et al [54] and Wong et al [55]. These policy actions converged with the physical distancing criteria and possible failures, presenting physical transmission isolation barriers for airborne transmission (aerosol-biosols and atmospheric conditions [20,35-39]). Chinazzi et al [56] discussed community policy actions regarding airplanes. At this point, a counter effect can be seen despite social physical distancing if social activities occur in outdoor spaces without the use of masks or city disinfection. Therefore, risk continues to be present.

Social connection might be one of the unobservable factors of transmission if the virus can spread under atmospheric conditions [35,36,57-60] and is still active in air fluids [20,35-39]. This would mean that a ground preventive framework is insufficient. Most of the recommendations for physical distancing issued during that time addressed the virus’s potential to spread on the ground and through the air via human bodily fluid droplets. Complex air-fluid scenarios without droplets involved (eg, pollution) were not considered. Wickramasinghe et al [57] reported several cases of person-to-person transmission patterns in that period, which can be understood as air transmission caused by the lack of virus social transmission isolation policies involving additional barriers, such as masks and city disinfection. Similar observations were made by Cembalest [58], based on a brief analysis, and by Pirouz et al [59], based on mathematical modeling with a deep analysis of how the atmospheric parameters of temperature, humidity, and wind affect the population density output for SARS-CoV-2 infection. These studies came to the proximal conclusion that atmosphere has a strong impact on the patterns of community virus dissemination in countries that adopted social physical distancing without mask policies and city disinfection. Finally, Poirier et al [60] examined the weather conditions capable of generating the full transmission patterns without a social transmission barrier for airborne transmission.

## Methods

The main goal of this paper is to identify the differences in outcomes among countries that adopted physical distancing measures in association with mask use and city disinfection during the period of analysis (January to April 2020). In this research, the social distancing framework without additional measures adopted by some countries represents the main model for the constant reproductive dissemination patterns of SARS-CoV-2 community transmission.

This paper takes an experimental approach to identify limitations in social distancing policy. Two groups of countries were selected. The first consisted of countries that adopted social distancing measures without specifying physical distancing, mask use, and city disinfection. The second consisted of countries that adopted all these measures between January and April 2020 (ie, only China and South Korea).

## Results

### Empirical Evidence for COVID-19 Transmission Instability

Table 1 presents the selected countries and their fluctuations in daily confirmed cases in random statistical data samples by date [31]. Countries marked with a superscripted “a” presented the best outcomes for daily new cases during the period investigated. The remaining countries in the other group presented inconsistent outcomes of daily new cases. This constitutes empirical evidence of instability in COVID-19 transmission in countries early in the pandemic.

**Table 1:** Rolling 3-day average of daily new confirmed cases of COVID-19 among selected countries from March 28-30, April 11-13, and May 1-2, 2020. Source: Our World in Data [31].

Country	Rolling 3-day average of new cases		
	March 28-30 (n=56,337)	April 11-13 (n=71,619)	May 1-2 (n=60,807)
United States	19,011	32,606↑	30,399↓
Spain	7536	5054↓	1149↓
Italy	5717	4283↓	1974↓
Germany	5003	4092↓	1354↓
France	3673	3914↑	1116↓
Iran	2968	1814↓	1020↓
United Kingdom	2621	6086↑	5436↓
Turkey	1863	4647↑	2579↓
Belgium	1534	1538↑	566↓
Switzerland	1187	703↓	147↓
Netherlands	1145	1288↑	458↓
Portugal	806	948↑	343↓
Canada	746	1342↑	1682↑
Austria	595	279↓	72↓
Brazil	447	1600↑	6567↑
Norway	315	103↓	51↓
Australia	309	79↓	9↓
Sweden	298	577↑	633↑
Denmark	173	198↑	153↓
China^a^	110	75↓	6↓
South Korea^a^	110	29↓	6↓
Finland	87	139↑	103↓
Singapore	83	225↑	716↑
Argentina	77	114↑	135↑
Chile	255	460↑	881↑
Saudi Arabia	101	367↑	1340↑
United Arab Emirates	45	359↑	552↑
Egypt	31	126↑	284↑
Pakistan	117	238↑	1076↑

^a^Presents the best outcomes of daily new cases during the period investigated.

### Maximum Exponential Growth and Epidemic Duration in Days

**Figure 1:**
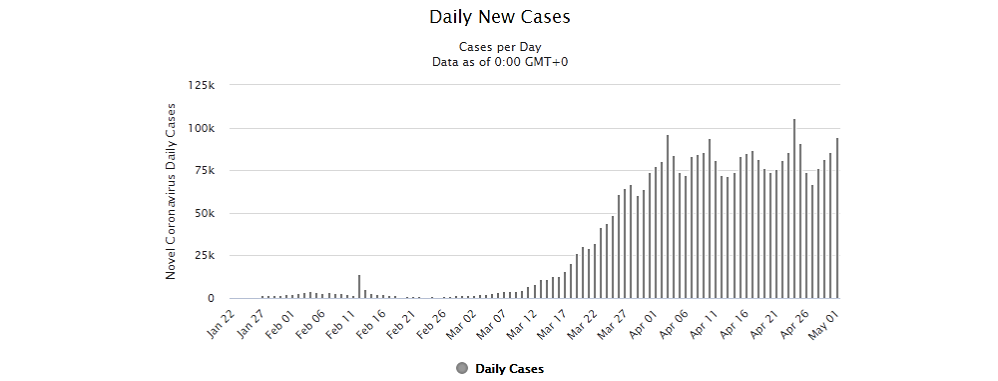
General overview of all reported cases of COVID-19 worldwide from February to May 2020. Source: Worldometer [61].

The statistical data in Figure 1 show the rise in daily new cases around the world, along with all policies adopted by countries, such as social distancing, COVID-19 testing, and physical distancing criteria, in association with (or without) the use of masks and city disinfection, from February to May 2020 [61].

Many European countries are adopting different measures for prevention. However, one specific point beyond social distancing and COVID-19 testing can be highlighted. As of April 2020, these countries had still not introduced mask use and/or constant city disinfection, which had been adopted by China and South Korea early in the pandemic and continued to be implemented later on. In late March and April [4], the infection rates in countries such as Italy, Spain, Iran, the United States, Germany, France, and Brazil were rising, with patterns different from those of China and South Korea (Table 1). This was still the case in May 2020.

In European countries [62], social distancing, COVID-19 testing availability, and physical distancing measures were introduced in late March and at the start of April. Although many citizens disobeyed institutional orders [63-66], reports indicated a reduced number of citizens outside their homes. However, daily infection cases were constantly over the population mean of 30,000 during April for a total of 58 days, from February 28 to April 25, 2020.

**Figure 2:**
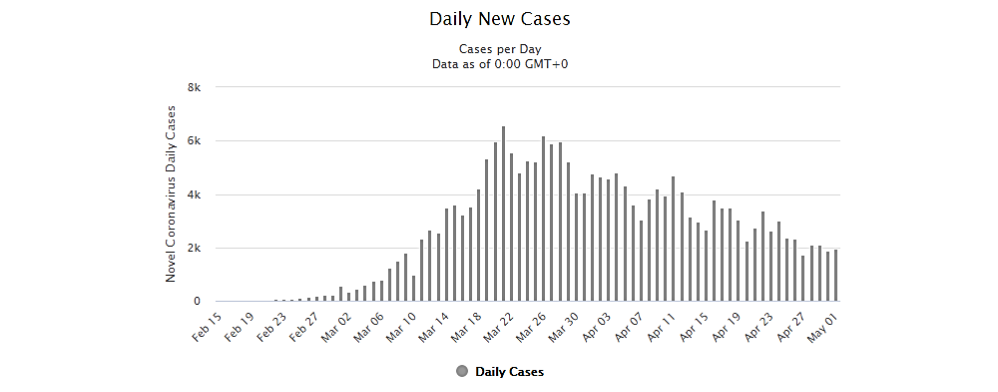
New daily cumulative COVID-19 cases in Italy. Note that Italy's mask use policy for the public was introduced by late March and early April, being this measure carried out until the last date this research was conducted. Source: Worldometer [61].

In Europe [62], and particularly in Italy (Figure 2), where individuals disobeyed orders to stay at home, these actions could have also generated several random transmission outputs. These specific random aspects contribute to the statistical variance of these countries, including the number of infected people and the mortality rate.

As shown in Figure 2, in Italy, the number of days of exponential growth represents constant daily infection cases with growing patterns, starting from the epidemic outbreak until a population mean of 4000 (maximum exponential growth rate for a period of 51 days, from February 22 to April 12, 2020).

Many other factors have been discussed to explain why virus spread was still rising in these countries, such as availability of testing and the date a city first implemented social physical distancing measures. Besides, it can also have a strong influence on the virus’ undetected phase of exponential growth; the time series of these statistical data also show how much time was needed for each country to stabilize its virus infection without the measures adopted by China and South Korea. Observing the preventive measures targeting airborne vectors (masks and city disinfection) that were adopted as default by China [67] and South Korea [68], the virus social transmission behavior differs from the other analyzed countries.

According to the data provided by Worldometer [67,68], these countries had adopted social physical distancing with air preventive measures, with a total of 20 days of maximum exponential growth rate over a population mean of 250 (February 19 to March 8, 2020) for South Korea and a total of 28 days of maximum exponential growth rate over a population mean of 1500 (January 23 to February 18, 2020) for China.

It is also important to consider exceptions for a possible microdimension of analysis of population biology that can occur in any country, as a local problem [69-71] does not always contribute to a high exponential growth rate of virus transmission. However, although the microdimensions were able to produce fluctuations in data, the whole scenario can be represented by descriptive statistics.

One other point concerning China is that its high exponential growth was due to the initial conditions of the new disease, and the country needed time to evaluate and adopt policies and scientific measures, as observed by Pan et al [71]. Additionally, China and South Korea adopted these measures early during their local epidemics based on their culture and experience with past epidemics; other countries were still trying to find alternative solutions at that time [63-66].

Compared to other countries, China and South Korea were the only true parameter of analysis of these policies. If we were interested in investigating any of the other numerous policies adopted by countries for any period of time, a country-by-country as well as a policy-by-policy analysis would be needed to check the results of each country’s policy. However, even without this kind of analysis, China and South Korea clearly presented the best scores for COVID-19 reduction during the period of interest.

### Maximum Exponential Growth Mean and Dissemination Rate Over Time

Table 2 shows the exponential growth patterns over time in China and South Korea. Data from other countries are also included, and the same data are presented in Figure 2 and in reference and news websites [61-68]. The first column of Table 2 presents the maximum growth of infection per population ratio obtained by the maximum exponential growth mean reached in an average day’s peak since the outbreak, and therefore does not account for growth above the mean *y* presented by some countries. This mean represents a critical value per population ratio reached by the infection, and it is counted if there is a positive exponential growth. If a second wave of infection is observed, it will count for this second period with a cumulative time since the outbreak. The second column *t* presents how many days the infection presented an exponential growth with a maximum mean reached. The third column contains the maximum exponential infection dissemination rate over days, following the theoretical design involving SIR models and missing gaps of this model for COVID-19.

The approach in the third column has similarities to SIR models, but it is based on distinct aspects of analysis of the variables S and R. These variables are removed from the formula, and the focus is mainly on variable I, defined by Weibull parameterizations and exponential distributions. This design of analysis has been very relevant due to the instability aspects of SIR analyses done since the disease outbreak, which occur mainly in the S and R compartments due to infodemics, uncertainty, the apparent lack of overall topological data homology, and other nonlinear aspects of COVID-19. For this reason, the proposed method of analysis considers only the infectious disease aspect of the evolution of cases, rather than assuming full immunity or using deterministic models for population behavior, which in this case is one of the most influential factors of propagating the virus.

For this analysis, it was assumed that the observed infected population samples *Y*=(*Y_1_*, …, *Y_n_*) experienced the exponential growth *f*(*Y*;*λ*)=*λe*^–^*^λY^*, where the samples were taken from zero cases to the observed maximum exponential growth mean reached per population ratio for each country, with an unknown predictive scale of *exp λ* or maximum likelihood estimator of *λ* due to the nonlinear outputs generated for *Y* with the heteroscedasticity form. In this simple form, where the mean is defined as *y*=1/*λ*, the numerical representation of the ratio between days and the mean can be obtained by observing the exponential mean scale until it reaches a form like *y*=*Y*, with *y* adopted for the calculations with the conditional shape of the Weibull parameterization like *κ*<1. At this point, the days counting forward in this condition are rejected to extract the maximum exponential infection dissemination rate according to the formula *R*=*y*⁄*t*, and *t*=*κ* only in the desired event expression. This approach can be more sensitive in terms of the progress of the disease over time and its potential to infect as time passes. This sensitivity leads to much more accurate predictions due to the exponential behavior of infections in the community phases of infection spread and dissemination patterns.

**Table 2:** COVID-19 maximum exponential growth patterns per population and time period by country or region from April 7 to May 1, 2020. The *y* and *t* data shown are for May 1, 2020. Data source: Worldometer [61].

Country	*y*	*t*	*R*		
			April 7	April 13	May 1
Worldwide^a^	60,000	99	675.67	886.07↑^b^	606.06↓^b^
Europe^a^	30,000	91	410.95	379.74↓	329.67↓
Italy	4000	51	90.90	78.43↓	78.43=
South Korea	250	20	12.5	12.5=^b^	12.5=
China	1500	28	53.57	53.57=	53.57=
Iran	2000	46	44.44	43.47↓	43.47=
Spain^a^	5000	55	131.57	121.95↓	90.90↓
France	4000	45	138.88	100↓	88.88↓
United States^a^	30,000	53	625	540.54↓	566.03↑
Brazil^a^	5000	51	25	28.57↑	98.03↑
Germany	4000	41	105.26	100↓	97.56↓
Russia^a^	5000	46	15.62	27.02↑	108.69↑
United Kingdom^a^	5000	58	64.51	69.64↑	86.20↑
Singapore^a^	500	57	0.96	1.75↑	8.77↑
Portugal	500	49	15.15	13.15↓	10.20↓
India^a^	1000	58	8.33	9.43↑	17.24↑
Canada^a^	1500	48	27.77	24.39↓	31.25↑
Japan^a^	500	67	3.92	7.14↑	7.46↑
Sweden^a^	500	58	5.71	0.65↓	8.62↑
Argentina^a^	100	57	1.51	2.56↑	1.75↓
Chile^a^	500	52	10	8.33↓	9.61↑
Saudi Arabia^a^	1000	54	3	4.16↑	18.51↑
United Arab Emirates^a^	400	70	4.34	5.76↑	5.71↓
Egypt^a^	200	62	1.31	2.27↑	3.22↑
Pakistan^a^	500	53	3.44	5.71↑	9.43↑

^a^Note that at the time of this writing, some countries were at their maximum exponential infection dissemination (different epidemic phases). For these countries, no final exponential score had been reached yet. However, this does not count for future predictions.

^b^↑, ↓, and = denote increase, decrease, and no change, respectively.

Note that in Table 2, some countries present a lower exponential growth rate than China or South Korea. These data need to be considered in the context of when the country’s outbreak started. Many countries were also at their maximum exponential growth at the time the data were collected. For these countries, it is not possible to judge whether their policies had already helped to flatten the curve of daily new cases, and some of them present active exponential growth; therefore, further future analysis is required to compare them to the other countries, as will be explained in the following paragraphs.

China, being the first country to adopt countermeasure policies, experienced some delay, and therefore, the maximum exponential rate was reached before these measures could take effect. In addition, many countries that had adopted measures based on previous experience performed better than the ones that were experiencing an epidemic for the first time. However, since they retained active low exponential growth (eg, Singapore, with a low maximum exponential rate), they did not reach the same results as China and South Korea with the adoption of additional preventive measures of social distancing/city disinfection and a high reduction of exponential virus spreading patterns. The Singapore scenario has occurred in many other countries as well. Singapore also presented a rise in the maximum exponential growth from 50 (April 7) to 500 (May 2). Germany, Italy, Portugal, Iran, and France presented a decline in the mean maximum exponential rate reached at the time the data were retrieved; however, this does not count for future epidemic behavior to be observed based on a deterministic approach.

Figure 2 and data from reference and news sources [61-68] show how long it took for some countries that implemented social physical distancing measures plus airborne transmission preventive methods to flatten the exponential growth of community infections. Countries that only applied social distancing of any sort without mask use or city disinfection at the early stages required many more days than other countries that applied airborne transmission prevention measures [63-66]. Many other scenarios were also observed since policies about mask use and city disinfection were still in the implementation phase in many countries.

It is also important to note that in Table 2 the data refer to different epidemic phases of data collection for each country. These distinct phases are important to consider together because a methodology is needed that can extract the behavior of the disease in the nonoptimal (deterministic) evolution of the virus infection and policies adopted by countries. This reveals a complex scenario involving the disease dynamics, a confounding environment, and possible convergence behavior of the policies adopted to mitigate the disease.

### Maximum Exponential Growth Mean × Time × Cases per 1 Million Population

Table 3 compares case counts in the selected countries on May 1, 2020 [61]. China and South Korea both have low case counts per 1 million population, low epidemic duration, and stable exponential growth. Notably, some countries present lower case counts per 1 million population, but they all have growing patterns of infection propagation, longer epidemic duration, and high exponential growth rate patterns. At the time of the analysis, China and South Korea had the best scores for the correlation between total cases per 1 million population over the period of infection and COVID-19 growth pattern stability. This is further evidence of the effectiveness of their policies. Note that any range of analysis to be performed will have its values of time and maximum exponential mean modified according to the selection taken. The higher the range, the better the *R* precision.

Even with good scores, some countries did not have optimal values for all the columns in Table 3 and presented an exponential growth rate, as of May 1, 2020. Although many of these countries are located close to China and South Korea, they do not match these countries’ later results; several factors influenced the oscillations and differences in the numbers. Notably, Argentina had the best score in South America and was ahead of many other regions worldwide. Voluntarily and later obligatory mask use and city disinfection took Argentina to the same epidemic scenario as China and South Korea, leading to successful results. The United Arab Emirates and Portugal, with their decreasing exponential growth rate, could reach better results by introducing air transmission preventive measures.

Table 3 clearly displays much of the unpredictability based on nonlinear factors such as the health policies adopted by each country, public health infrastructure, population genetics, COVID-19 testing availability, and citizens’ adherence to social distancing of any type. These data indicate that further studies are still necessary to obtain more accurate numerical results, since each country undergoes a period of disease dissemination with different rates. Although these variances produce large differences in outcomes, most countries adopted social distancing as a method of virus spread prevention, with no obligation of social physical distancing, which became a default pattern for prevention in late February and early March. This also contributed to the virus incubation period and caused the dissemination rates to increase much more than in China and South Korea. These results point to the conclusion that while many factors influence outcomes, some specific patterns occur only in these two countries and in none of the others. By April 30, 2020, China and South Korea had shorter epidemic durations than other countries, stable low disease exponential growth patterns, and low confirmed case counts per 1 million population [14].

Table 4 extends this analysis to the period from May 1 to June 2, 2020.

Between May 1 and June 2, out of the 25 countries analyzed, 11 presented differing infection dissemination patterns, while 14 had a constant evolution of infection that also indicates a positive analysis for the predictive statistics, despite the long period of time considered (sensitivity and prediction for 33 days).

The analysis shows that prediction for a shorter or longer time frame is highly associated with the type of policies adopted by the selected countries as compared to China and South Korea. China and South Korea still had the best results for local epidemic reduction. Notably, Spain and Italy reached a stable point in transmission during the period of analysis through lockdown measures rather than mask use or city disinfection. However, while lockdowns helped them reach the same status as China and South Korea, these policy measures worked differently. The first difference is the time it took to reach stability. For China and South Korea, it was approximately 28 and 20 days, respectively. On the other hand, Spain and Italy took 55 and 51, respectively. While the lockdown was active and no mass mask use was mandatory, the time it took to reach the peak and flatten the curve was higher in these countries. Resurgences of infection also occurred, and it was difficult to reach a very low mean of daily new cases after the curve was flattened [61]. This suggests that lockdown measures alone were not enough to flatten the curve to the level of China and South Korea. Gradually, these countries, as well as many others, started to use masks and carry out city disinfection in May, June, and July 2020.

**Table 3:** Countries with COVID-19 dissemination and total infected cases per 1 million population, as of May 1, 2020. Data source: Worldometer [61].

Country	Total cases, N	Total cases per 1 million population	*t*	*R* on May 1
United States	1,159,430	3503	53	566.03↑^a^
Italy	209,328	3462	51	78.43=^a^
China^b^	82,875	58	28	53.57=
Spain	245,567	5252	55	90.90↓^a^
Germany	164,967	1969	41	97.56↓
Iran	96,448	1148	46	43.47=^c^
France	168,396	2580	45	88.88↓
United Kingdom	182,260	2685	58	86.20↑
Sweden	22,082^c^	2186	58	8.62↑^c^
India	39,699^c^	29^c^	58	17.24↑^c^
Japan	14,305^c^	113^c^	67	7.46↑^c^
South Korea^b^	10,780	210	20	12.50=
Russia	124,054	850	46	108.69↑
Singapore	17,548^c^	2999	57	8.77↑^c^
Portugal	25,190^c^	2470	49	10.20↓^c^
Canada	56,714^c^	1503	48	31.25↑^c^
Brazil	96,559	454	51	98.03↑
Argentina	4532^c^	100^c^	57	1.75↓^c^
Chile	18,435^c^	964	52	9.61↑^c^
Saudi Arabia	25,459^c^	731	54	18.51↑^c^
United Arab Emirates	13,599^c^	1375	70	5.71↓^c^
Egypt	6193^c^	61^c^	62	3.22↑^c^
Pakistan	19,022^c^	86^c^	53	9.43↑^c^

^a^↑, ↓, and = denote increase, decrease, and no change, respectively.

^b^Indicate the best scores reached by China and South Korea.

^c^Indicates countries that reached the best score compared to China and South Korea.

**Table 4:** COVID-19 maximum exponential growth patterns per population and time period by country or region from May 1 to June 2, 2020. Data source: Worldometer [61].

Country	*R*	
	May 1	June 2
Worldwide	606.06↓^a^	572.51↓
Europe	329.67↓	162.60↓
Italy	78.43=^a^	78.43=
South Korea^b^	12.50=	0.09↓^a^
China	53.57=	53.57=
Iran^b^	43.47=	25.64↓
Spain	90.90↓	90.90=
France	88.88↓	3.89↓
United States^b^	566.03↑	235.29↓
Brazil	98.03↑	180.72↑
Germany	97.56↓	2.73↓
Russia^b^	108.69↑	96.15↓
United Kingdom^b^	86.20↑	22.22↓
Singapore^b^	8.77↑	5.61↓
Portugal	10.20↓	1.85↓
India	17.24↑	64.44↑
Canada^b^	31.25↑	9.37↓
Japan^b^	7.46↑	0.30↓
Sweden^b^	8.62↑	6.66↓
Argentina^b^	1.75↓	6.17↑
Chile	9.61↑	41.66↑
Saudi Arabia^b^	18.51↑	17.44↓
United Arab Emirates	5.71↓	5.39↓
Egypt	3.22↑	7,.44↑
Pakistan	9.43↑	23.52↑

^a^↑, ↓, and = denote increase, decrease, and no change, respectively.

^b^Countries that presented a different behavior of infection dissemination compared to that observed on May 1, 2020, the start date of the analysis.

## Discussion

### Principal Findings

The nonlinear aspects and variables of COVID-19 transmission and prevention require multiple factors to be considered, such as health infrastructure facilities, new design of workflows/structures to prevent infection in health facilities, type and availability of personal protective equipment, public health policies adopted by each country, population genetics, COVID-19 testing availability and rapid response, social distancing, economic activities in some essential and nonessential sectors, government policies for supporting the population and survivability, citizens’ collaboration with policies, and other public health and social policies. We did not aim to produce statistical numerical results involving all these variables, due to the likely lack of significance of data correlation (heteroscedasticity) for demonstrating that the results presented in this paper are due only to the selected type of policy interventions. All the nonlinear aspects mentioned affect epidemics in different ways. However, we focused on three aspects: the amount of time that has passed since the infection has occurred; what the maximum infected population range was; and how many people per million have been infected. These questions address specific preventive measures, and in this context, the type of policy analyzed can be considered the main countermeasure. Therefore, statistical analysis with numerical results is unlikely to provide any important information about community transmission in terms of seasonality due to the limited time period for which the data were available and to the nonlinear properties of the variables necessary for predicting daily new virus cases in each country. For this reason, the influence of policies on daily new cases was roughly described by filtering out other factors that were unlikely to accommodate the nonlinear scenario of the disease. The results show that policies directly affected the population; they can also influence many of the nonlinear sets of variables described earlier (a convergence aspect of higher-order nonautonomous functions).

**Figure 3:**
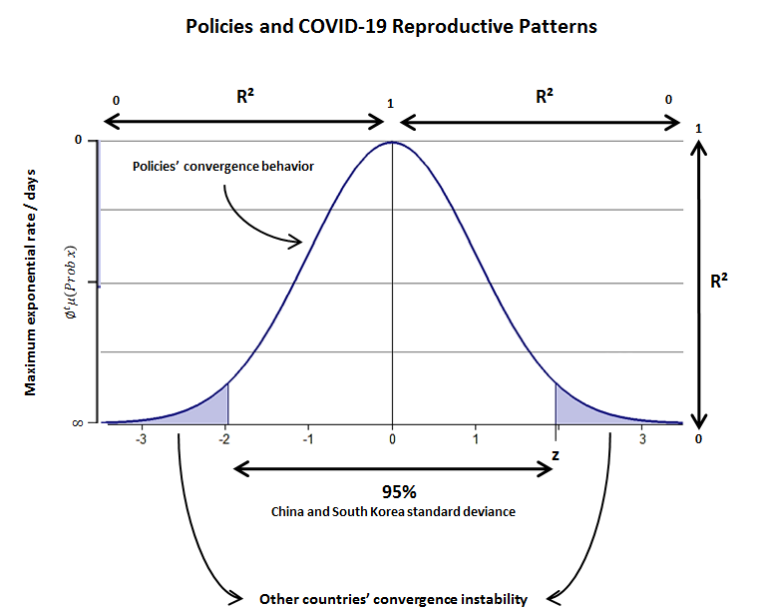
Representative scheme of SARS-CoV-2 reproductive patterns among countries whose policies might or might not converge toward a very low maximum exponential rate of infection per population/days. Note that countries with a low maximum exponential rate (Table 2) also present active infection patterns, with this feature being a nonconvergence of the type of policies adopted and, hence, expressing an exponential probability of infection constant growth (false null hypothesis).

However, an overview of the nonparametric data was provided to assess the types of policies investigated in this research for a seasonal forcing behavior with a strong influence on the overall scenario. While this research did not focus on statistical numerical results for all relevant variables, these inferences were done in terms of the conceptualization of *z* and *P* value tests, SD, variance analysis, and linear regression analysis of the policies in selected countries, as shown schematically in Figure 3.

Figure 3 shows that the nonlinear behavior of COVID-19, with preventive policies as mandatory measures to be adopted. Although many policies do not stop virus dissemination entirely at the minimum rate, the results demonstrate that China’s and South Korea’s policies might be more successful at keeping the virus exponential growth at a low rate.

The COVID-19 event was analyzed from a theoretical point of view using the qualitative theory of differential equations framework to understand how the input of many variables and output in terms of convergence and stability of the policies adopted by each country could yield visible differences in daily new cases and maximum time for exponential infection growth. The results show true differences between the policies adopted and the parameters mentioned earlier; however, future studies from this point of view are needed.

Furthermore, while the variance observed in daily new cases among countries over the period of interest was produced by different factors in each country, points of convergence (the policy type fixed-point theoretical approach) are considered stable from a policy analysis point of view and have high stability (COVID-19 reduction) in many solutions obtained from the confounding environment. Even with high variance produced by other variables that influence COVID-19 transmission, these fixed-point stable parameters can create a confidence region of statistical analysis by reducing the maximum exponential growth of the virus over time; therefore, it could be more conclusive than many mathematical infectious disease models (SIR stochastic or deterministic approaches) developed since the beginning of the epidemic and later pandemic dissemination. Official, preassumed forms of social physical distancing measures were adopted to avoid COVID-19 transmission during that time, and the possible new patterns of atmospheric disease transmission may constitute a previously unobserved, continuous (not discretized) form of transmission (partially unpredictable) due to airborne instability properties. These time-varying, unresolved empirical data have been presented roughly, since this paper evaluated the entire epidemic scenario with aggregated data.

These results, from January to April 2020 [72], demonstrates that even 20 infected individual hosts can constitute a risk of propagating the disease [48]. This was observed by the end of March in China and Japan when the policies adopted by successful countries were eased. Nevertheless, the statistical data presented in this research strongly suggest that social distancing fails in some countries, but succeeds in others because of the additional use of masks and city disinfection.

The asymptotic instability aspect of the statistical data in Figure 2, as well as data from internet sources [61-68], yields lower infection rates for some countries (China and South Korea) and exponential infection rates for others. This can be explained as the virus asymptote transmission behavior of the emergent phenomenon [35-39,73,74] caused by community behavior [75] based on social distancing failures in most of the countries, while the use of masks and city disinfection in China and South Korea yielded the best results in reducing disease spread and dissemination patterns.

While this research was being conducted, the daily new cases in European countries started declining (March 31, 2020). This can be attributed to the effect of the social physical distancing policy. However, China and South Korea used different measures based on previous experience. The maximum range of infection reduction with only social physical distancing is limited, since many workforce sectors are still active. Therefore, this research suggests that active citizens should use masks [75], and countries should start to disinfect public spaces, including public transport vehicles and routes. These measures will require the introduction of policies to relax the lockdown in cities by strategically and gradually allowing the population outside their homes with additional new social distancing preventive methods.

Digital behavior (infodemics) [76,77] was not considered here, despite its potentially high influence on virus transmission due to misinformation and misuse of scientific information. This is a limitation of this research, since even if a country has adopted all the necessary measures, its citizens can undermine it. This factor should be considered case by case, and it does not significantly contradict the results.

### Conclusions

This study theoretically and empirically investigated preventive measures in different countries; the results show that virus transmission patterns are closely linked with human social behavior and the environmental airborne transmission of SARS-CoV-2. Therefore, countries should adopt preventive policy measures and control individual behavior.

Countries that adopted policy measures based on evidence of the atmospheric transmission of COVID-19 reported shorter local epidemic duration, fewer cases per 1 million population, a lower maximum exponential growth mean rate per population, and a lower rate of the COVID-19 daily new cases over time.

Looking at policy measures holistically, social physical distancing and COVID-19 testing availability are mandatory for any country’s policy since they are the most reliable and convergent ways to reduce community virus transmission and flatten the curve. Concerning the transmission isolation observed in China and South Korea and the superspreading patterns observed in other countries from January to April 2020, the results show full convergence of nonlinear variables for higher virus infection reduction affecting the input-output of SARS-CoV-2 propagation over time with the adoption of COVID-19 testing availability and social physical distancing by 1- 2 m, along with the additional use of masks and sanitization (city disinfection). Remarkably, China and South Korea adopted these policy measures early in the pandemic, in contrast to other countries. Due to these measures, China and South Korea obtained better results in controlling the local epidemic.

The results observed in South Korea are consistent with those of China. Other countries that did not follow use masks or perform city disinfection presented high nonlinear outputs of SARS-CoV-2 transmission; a common feature for these countries was the constant growth in new infection cases day by day even with the use of social physical distancing measures. This observation suggests that the virus can be transmitted beyond the recommended distance of 1 or 2 m. This was confirmed by Liu et al [15] in April 2020 and by Morawska et al [3] in July 2020. The use of masks and city disinfection appears to be the best strategy for reducing SARS-CoV-2 spread patterns (forms of transmission) and dissemination patterns early in the worldwide pandemic.

Another important point is that if COVID-19 testing is not fully available, social physical distancing measures along with the use of masks and city disinfection can help prevent spread, since they help to isolate undetected infected individuals (including asymptomatic cases), prevent airborne transmission, and protect uninfected people from environmental transmission.

While this research was being conducted in April and early May, some European countries analyzed in this study implemented city disinfection, mask use, and lockdowns, which likely helped to reduce the airborne transmission of SARS-CoV-2. In addition, in Brazil, the most basic physical distancing policy was ignored by many citizens and publicly ignored by the country’s president. This may be why Brazil had the third highest number of confirmed COVID-19 cases in the world on September 9, 2020, and has, as of March 2021, a mean rate of more than 2500 COVID-19 deaths daily.

## Abbreviations

MERS: Middle Eastern respiratory syndrome
SARS: severe acute respiratory syndrome
SIR: susceptible, infected, removed
TCID: tissue culture infective dose
