# Correction: The Impact of COVID-19 Management Policies Tailored to Airborne SARS-CoV-2 Transmission: Policy Analysis

**DOI:** 10.2196/30007

**Published:** 2021-05-03

**Authors:** Charles Roberto Telles, Archisman Roy, Mohammad Rehan Ajmal, Syed Khalid Mustafa, Mohammad Ayaz Ahmad, Juan Moises de la Serna, Elisandro Pires Frigo, Manuel Hernández Rosales

**Affiliations:** 1 Internal Control Center Secretary of State for Education and Sport of Paraná Curitiba Brazil; 2 Mathematics Group Department of Physics, Faculty of Science Institute of Science, Banaras Hindu University Varanasi India; 3 Department of Biochemistry Faculty of Science University of Tabuk Tabuk Saudi Arabia; 4 Department of Chemistry Faculty of Science University of Tabuk Tabuk Saudi Arabia; 5 Department of Physics Faculty of Science University of Tabuk Tabuk Saudi Arabia; 6 Universidad Internacional de la Rioja Madrid Spain; 7 Federal University of Paraná Curitiba Brazil; 8 Programa Universitario de Estudios Sobre la Ciudad National Autonomous University of Mexico Mexico City Mexico

In “The Impact of COVID-19 Management Policies Tailored to Airborne SARS-CoV-2 Transmission: Policy Analysis” (JMIR Public Health Surveill 2021;7(4):e20699), the authors noted one error.

The academic degree of author Archisman Roy has been corrected from “MSc” to “BSc.”

The correction will appear in the online version of the paper on the JMIR website on May 3, 2021, together with the publication of this correction notice. Because this was made after submission to PubMed, PubMed Central, and other full-text repositories, the corrected article has also been resubmitted to those repositories.

